# Association of Mandatory Bundled Payments for Joint Replacement With Use of Postacute Care Among Medicare Advantage Enrollees

**DOI:** 10.1001/jamanetworkopen.2019.18535

**Published:** 2019-12-27

**Authors:** David J. Meyers, Cyrus M. Kosar, Momotazur Rahman, Vincent Mor, Amal N. Trivedi

**Affiliations:** 1 Department of Health Services, Policy, and Practice, Brown University School of Public Health, Providence, Rhode Island; 2 Providence VA Medical Center, Providence, Rhode Island

## Abstract

**Importance:**

In 2016, the Centers for Medicare & Medicaid Services introduced mandatory bundled payments for knee and hip replacement surgical procedures among traditional Medicare (TM) patients in randomly selected areas. The association of bundled payments with outcomes among patients enrolled in Medicare Advantage (MA) is not known.

**Objective:**

To determine the association of bundled payments for joint replacement surgical procedures with the use of postacute care (PAC) services among MA patients.

**Design, Setting, and Participants:**

This cohort study used difference-in-differences analysis to evaluate changes in PAC use among patients enrolled in Medicare who underwent joint replacement operations before and after the introduction of bundled payments (ie, from January 1, 2013, to September 30, 2017). A total of 75 metropolitan statistical areas were randomized to participate in the bundled payment program, with 121 areas serving as controls. Data were analyzed between September 15, 2018, and October 1, 2019.

**Exposure:**

Bundled payments for hip and knee joint replacement operations, in which hospitals received a single payment to cover all costs associated with a joint replacement and associated care for the 90 days after surgery.

**Main Outcomes and Measures:**

The primary outcomes were discharge to any institutional PAC setting and days spent in institutional PAC within 90 days after surgery. Secondary outcomes included discharge and days spent in specific PAC settings (ie, home health, skilled nursing facility, inpatient rehabilitation).

**Results:**

Of 1 536 387 individuals who underwent hip and knee join replacement surgery, 493 977 (32.2%) were enrolled in MA (mean [SD] age, 73.3 [8.4] years; 386 699 [63.5%] women; 55 078 [6.4%] black) and 1 042 410 (67.8%) were enrolled in TM (mean [SD] age, 73.3 [8.7] years, 829 014 [65.2%] women; 82 890 [9.4%] black). Among MA patients, bundled payments were associated with a reduction of 1.5 (95% CI, 1.0-2.0) percentage points in discharge to an institutional PAC setting (*P* < .001) and an estimated reduction of 0.3 (95% CI, 0.2-0.5) days spent in an institutional PAC setting (*P* < .001), a 5.6% relative reduction. Among TM patients, bundled payments were associated with a reduction of 2.6 (95% CI, 2.2-2.9) percentage points in institutional PAC discharge (*P* < .001) and a reduction of 0.8 (95% CI, 0.7-0.9) days spent in an institutional PAC setting (*P* < .001), a 2.5% relative reduction. These changes were larger in hospitals with greater proportions of TM patients. In hospitals with low concentrations of MA patients, time spent in institutional PAC settings decreased by 0.9 days among TM patients and 0.8 days among MA patients; in hospitals with high MA concentrations, time spent in institutional PAC settings decreased by 0.6 days for TM patients and 0.2 days for MA patients.

**Conclusions and Relevance:**

In this study, the first 18 months of the Centers for Medicare & Medicaid Services bundled payment program for joint replacement surgery were associated with reductions in the use of institutional PAC among MA patients. Past evaluations of bundled payments that focused on TM patients may not have measured the full consequences of this alternative payment model.

## Key Points

**Question:**

Was the Comprehensive Care for Joint Replacement (CJR) program associated with changes among patients enrolled in Medicare Advantage plans, privately managed care plans that insure 34% of Medicare patients and were not participants in the CJR program?

**Findings:**

In this cohort study of more than 1.5 million patients, the CJR program was associated with a 5.6% reduction in institutional postacute care days among traditional Medicare patients and a 2.5% reduction for Medicare Advantage patients, indicating that the policy change may have affected more patients than previously anticipated.

**Meaning:**

Alternative payment models in traditional Medicare may affect care in the Medicare Advantage program; therefore, evaluations of the CJR program that exclude Medicare Advantage may not capture the full consequences of the policy.

## Teaser

This cohort study determines the association of bundled payments for joint replacement surgical procedures with the use of postacute care services among patients enrolled in Medicare Advantage.

## Introduction

In 2016, the Centers for Medicare & Medicaid Services (CMS) launched the Comprehensive Care for Joint Replacement (CJR) mandatory bundled payment program to promote more efficient care for common joint replacement surgical procedures.^1^ Under this payment model, CMS prospectively pays hospitals for the initial surgery and all care covered by Medicare Parts A and B during the 90 days after surgery. Participating hospitals received bundled payments for all knee and hip joint replacement episodes among patients enrolled in traditional Medicare (TM). The Centers for Medicare & Medicaid Services required all hospitals located in randomly selected areas to participate in the CJR model, allowing for rigorous assessment of its effects.

Prior evaluations of the CJR program have reported reductions in postacute care (PAC) use and spending after the start of the program without evidence of increased surgical complications or changes in the proportion of operations performed on patients with high risk.^2,3^ These studies have generally focused on TM patients. However, bundled payments may catalyze changes in hospitals’ care practices for all patients, suggesting the possibility of spillover effects to joint replacement care financed by other payers.^4^ Furthermore, because bundled payments only apply to TM patients, hospitals with lower proportions of such patients may be less responsive to this payment policy change. A prior study^5^ found evidence of an association of the CJR program with reductions in discharge to institutional PAC among Medicare Advantage (MA) patients, but the study included a limited 6-month follow-up period and did not assess the use of PAC services in the 90 days after joint replacement surgery.

In this study, we first assessed the association of the CJR program with PAC use among patients in MA plans, privately managed care plans that insure 34% of Medicare beneficiaries and were not participants in the CJR program. We also investigated whether the associations of the CJR program with PAC use differed among hospitals with greater proportions of MA patients.

## Methods

### Data Sources

We acquired the Medicare Provider Analysis and Review (MedPAR) files from January 1, 2013, to September 30, 2017, which include hospital claims from all TM and MA patients treated in hospitals that receive Disproportionate Share Hospital or medical education payments.^6^ Such hospitals account for approximately 90% of Medicare hospitalizations^6,7^ and 90% of the joint replacement surgical procedures that were conducted in treatment and control metropolitan statistical areas (MSAs) in our sample. We assessed patient demographic characteristics and MA enrollment status on the month of hospital admission with data from annual Medicare Master Beneficiary Summary files. We also obtained patient assessment data collected in 3 organized PAC settings. These included the Minimum Data Set for skilled nursing facility (SNF) stays, the Inpatient Rehabilitation Facility Patient Assessment Instrument for inpatient rehabilitation facility (IRF) stays, and the Outcome and Assessment Information Set for home health visits. The Centers for Medicare & Medicaid Services requires all Medicare- or Medicaid-certified SNFs, IRFs, and home health care agencies to collect assessment data until patients are discharged, regardless of insurance status. Each assessment file was available through 2017, with the exception of the Outcome and Assessment Information Set for home health care, which was available through 2016.

We combined claims and PAC assessment data using the residential history file methodology,^8^ which enabled us to track patients’ locations on each day of the 90-day postsurgical period. While MedPAR contains a discharge status code that indicates type of PAC setting, our analysis suggested that this variable lacks accuracy (eTable 1 in the Supplement). Mandatory PAC assessments likely provide a more valid source of discharge location.

Brown University’s institutional review board approved the study protocol and waived the need for informed consent owing to the infeasibility of acquiring consent in claims data. The study followed the Strengthening the Reporting of Observational Studies in Epidemiology (STROBE) reporting guideline.

### Intervention Design

Under the CJR bundle, CMS set annual benchmarks for spending on all care for patients from the date of surgery until 90 days after surgery. If hospitals spent more than the benchmark, they paid a penalty to CMS. If hospitals spent less than the benchmark, they participated in the shared savings.

In July 2015, CMS announced the 75 MSAs that were randomly chosen to participate in the bundle, with 121 randomly selected control MSAs. The randomization divided MSAs into groups based on historical joint replacement spending, and MSAs with higher spending had a higher probability of selection. A total of 8 MSAs were excluded from the treatment group in October 2015. Similar to prior evaluations of the study,^2^ we included all 75 MSAs in our analysis in an intent-to-treat design. If a hospital was located within a chosen MSA, all procedures for hip and knee joint replacement (Medicare Severity Diagnosis Related Group codes, 469 and 470) among TM patients were subjected to bundled payment. Mandatory bundled payments officially began on April 1, 2016.

### Study Population

Our analyses included all TM and MA patients hospitalized with a Medicare Severity Diagnosis Related Group code of 469 or 470 in any treatment or control MSA from January 1, 2013, to September 30, 2017. We assessed the association of the CJR program with PAC use before and after 2 points. The first was July 14, 2015, when the hospitals that would participate were announced. The second was April 1, 2016, when mandatory bundled payments were implemented.

### Variables

Our primary outcome was the number of days in the 90-day postsurgery period that each patient spent in an institutional PAC setting (ie, an IRF or SNF). We focused on this outcome because prior evaluations have found that the CJR program led to changes in PAC use, and decreases in institutional PAC use were associated with the largest changes in spending. Secondary outcomes were discharge to any institutional PAC setting, estimated total spending, initial hospital length of stay, discharge to home with and without home health care, and the number of days in each posthospital setting.

We approximated spending for MA patients using average spending on TM patients because MedPAR lacks MA plan reimbursements. Specifically, we calculated the mean Medicare expenditure per day in each PAC setting for all TM patients in the sample. We then multiplied these estimates by the number of days MA patients spent in each location. Given that MA pays different rates than TM for most services, this analysis provided an estimate of spending among the MA population if average reimbursements were equivalent to those under TM. We did not include any reconciliation payments that hospitals may have paid back to CMS for exceeding the value of the bundle in these calculations.

### Statistical Analysis

We first compared patient characteristics between the treatment groups, the pre-CJR and post-CJR periods, and MA enrollment status. We then plotted quarterly trends in each outcome by treatment and MA status to descriptively assess changes over time.

For our primary analysis we used a difference-in-differences (DiD) framework to estimate the mean change in treatment associated with bundled payments. All primary analysis was conducted using an episode-level analytic file. We first used a Poisson model without fixed effects to compare differences in the number of joint replacement episodes over time. Next, we fit linear regressions for the other outcomes of interest, adjusting for sex, race/ethnicity, Medicaid eligibility, quarter, and hospital fixed effects. The inclusion of hospital fixed effects allowed us to compare outcomes within hospitals before and after the CJR program. Each model included an indicator for treatment assignment, an indicator for pre-CJR or post-CJR period, and their interaction, which was the DiD estimate. All models were stratified by MA status. In all analyses, we weighted episodes in each MSA by the probability that the MSA was selected in the randomization process, a method that has been used in prior evaluations.^2^ To test whether the outcomes among MA patients were significantly different from those observed among TM patients, we also fit triple difference-in-differences models (eTable 2 in the Supplement).

As the announcement of the CJR model took place 9 months before the start of financial penalties, hospitals may have begun changing practices before April 2016. To test this, we fit models with an alternative post-CJR period starting with the announcement of CJR randomization.

As the CJR program was targeted to TM patients, hospitals with a greater percentage of TM patients may have been at greater financial risk of CJR penalties. To see if this could lead to differences in outcomes, we stratified all analyses by each hospital’s proportion of Medicare patients receiving joint replacements who were enrolled in MA.

To ensure the validity of the DiD approach, we assessed whether pre-CJR trends in outcomes among treatment and control MSAs were parallel (eTable 3 in the Supplement). We also tested alternative specifications using different covariates, with and without probability weights, and with hospital random effects. Data were collected and analyzed between September 15, 2018, and October 1, 2019. A 2-tailed *P* < .05 was the threshold for statistical significance. All analysis was conducted using Stata version 15 (StataCorp).

## Results

The study population of 1 536 387 individuals included 493 977 patients (32.2%) enrolled in MA (mean [SD] age, 73.3 [8.4] years; 386 699 [63.5%] women; 55 078 [6.4%] black) and 1 042 410 (67.8%) enrolled in TM (mean [SD] age, 73.3 [8.7] years; 829 014 [65.2%] women, 82 890 [9.4%] black). A total of 603 965 patients (57.9%) with TM and 275 239 patients (55.7%) with MA underwent joint replacement operations in the pre-CJR period, and 438 445 patients (42.1%) with TM and 218 738 patients (44.3%) with MA underwent joint replacement operations in the post-CJR period. Across all available demographic characteristics, patients did not differ substantially between treatment and control MSAs or between pre-CJR and post-CJR groups (Table 1).

**Table 1.** Characteristics of Traditional Medicare and Medicare Advantage Patients Receiving Hip or Knee Replacement in Bundled Payment and Control MSAs

Characteristic	No. (%)							
	Traditional Medicare				Medicare Advantage			
	Bundled Payment MSA		Control MSA		Bundled Payment MSA		Control MSA	
	Pre-CJR^a^	Post-CJR^b^	Pre-CJR^a^	Post-CJR^b^	Pre-CJR^a^	Post-CJR^b^	Pre-CJR^a^	Post-CJR^b^
No.	269 723 (100)	195 520 (100)	334 242 (100)	242 925 (100)	126 402 (100)	99 130 (100)	148 837 (100)	119 608 (100)
Age, mean (SD), y	73.4 (8.8)	73.4 (8.5)	73.2 (8.8)	73.1 (8.5)	73.4 (8.4)	73.5 (8.2)	73.2 (8.3)	73.2 (8.2)
Dually eligible^c^	30 755 (11.4)	21 391 (10.9)	34 842 (10.4)	24 704 (10.2)	17 628 (13.9)	14 498 (14.6)	17 280 (11.6)	14 685 (12.3)
Women	171 882 (63.7)	124 211 (63.5)	212 213 (63.5)	153 870 (63.3)	82 729 (65.4)	65 067 (65.6)	96 233 (64.7)	77 765 (65.0)
Elixhauser comorbidities, mean (SD), No.^d^	2.4 (1.7)	2.4 (1.6)	2.3 (1.6)	2.3 (1.6)	2.4 (1.6)	2.5 (1.7)	2.4 (1.6)	2.4 (1.6)
Race/ethnicity								
White	340 938 (88.6)	171 800 (87.9)	429 906 (89.9)	216 604 (89.2)	145 294 (84.2)	81 833 (82.6)	176 190 (87.1)	102 152 (85.4)
Black	24 683 (6.4)	12 470 (6.4)	30 314 (6.3)	15 419 (6.4)	16 093 (9.3)	10 079 (10.2)	17 612 (8.7)	11 289 (9.4)
Asian	4518 (1.2)	2438 (1.3)	3208 (0.7)	1725 (0.7)	2296 (1.3)	1416 (1.4)	1465 (0.7)	978 (0.8)
Hispanic	5322 (1.4)	2571 (1.3)	4725 (1.0)	2362 (1.0)	4626 (2.7)	2919 (2.9)	2968 (1.5)	2207 (1.9)
Native American or American Indian	1029 (0.3)	551 (0.3)	1792 (0.4)	947 (0.4)	271 (0.2)	170 (0.2)	501 (0.3)	247 (0.2)
Other or unknown	8141 (2.1)	5690 (2.9)	8495 (1.8)	5868 (2.4)	4077 (2.4)	2713 (2.7)	3536 (1.8)	2735 (2.3)
Diagnosis related group code								
469, Major joint replacement with MCC	14 344 (5.3)	10 176 (5.2)	16 680 (5.0)	11 841 (4.9)	5889 (4.7)	4655 (4.7)	6813 (4.6)	5296 (4.4)
470, Major joint replacement without MCC	25 5379 (94.7)	185 344 (94.8)	317 562 (95.0)	231 084 (95.1)	120 513 (95.3)	94 475 (95.3)	142 024 (95.4)	114 312 (95.6)

Abbreviations: CJR, Comprehensive Care for Joint Replacement; MCC, major complications or comorbidities; MSA, metropolitan statistical area.

^a^ From 2013 to quarter 1 of 2016.

^b^ Quarter 2 and 3 of 2016.

^c^ Represents those who are dually eligible with Medicaid.

^d^ Based on diagnosis codes from Medicare Provider Analysis and Review Files during hospitalization for joint replacement operation alone.

**Figure 1.**
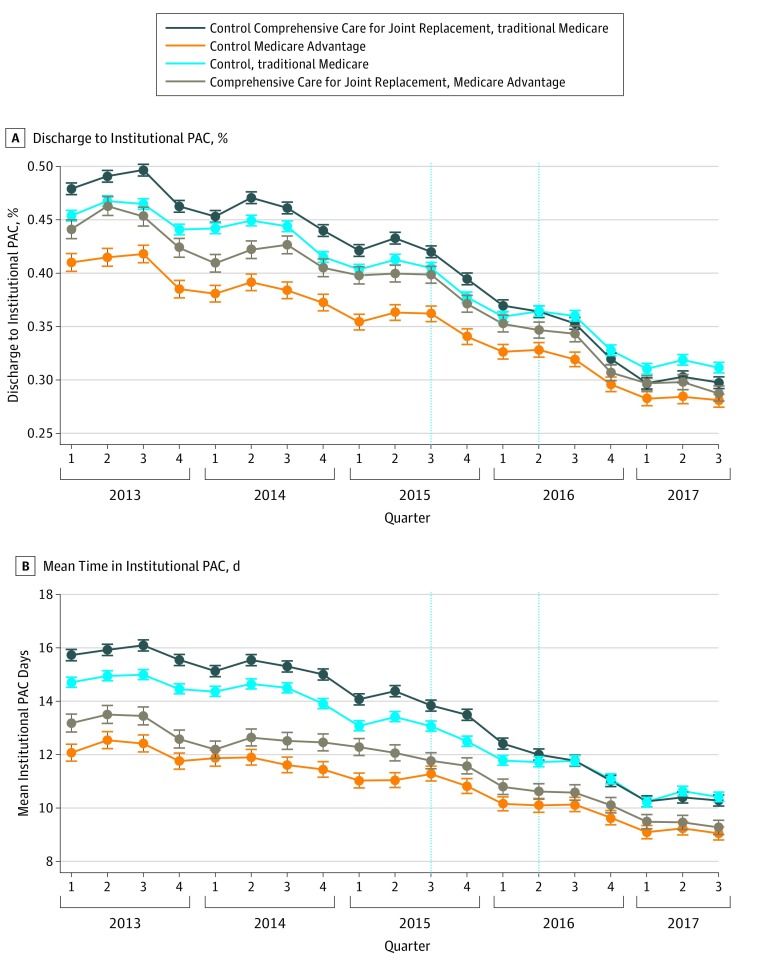
Trends in Days Spent in Institutional Postacute Care (PAC) Setting Institutional PAC settings include discharge to and days spent in skilled nursing facilities or inpatient rehabilitation facilities. The first vertical line represents the announcement of the Comprehensive Care for Joint Replacement program. The second vertical line represents the start of financial incentives for the Comprehensive Care for Joint Replacement program. Lines are unadjusted for patient characteristics.

In Figure 1, we plotted trends in the proportion of discharges to an institutional PAC setting and the number of days spent in institutional PAC settings for MA and TM control and treatment groups. In both outcomes, there was a steady decrease in institutional PAC use throughout the study period. At the beginning of the study period, the discharge to PAC rate was 45.4% among TM control patients, 47.9% among TM treatment patients, 41.1% among MA control patients, and 44.1% among MA treatment patients. These rates decreased during the study period to 31.1%, 29.8%, 28.1%, and 28.7%, respectively. Likewise, at the beginning of the study, mean time in institutional PAC was 14.7 days among TM control patients, 15.7 days among TM treatment patients, 12.1 days among MA control patients, and 13.2 days among MA treatment patients. Mean time in institutional PAC decreased to 10.4 days, 10.3 days, 9.1 days, and 9.3 days, respectively. The decrease seemed to accelerate after the CMS announcement of randomization and final rules for the CJR program in July 2015. For example, the unadjusted difference in time spent in an institutional PAC setting between the announcement of the CJR program and its implementation was 1.6 days among TM control patients, 1.8 days among TM treatment patients, 0.8 days among MA control, and 1.4 days among MA treatment patients. We present these figures for all other outcomes in the eFigure in the Supplement.

Table 2 presents unadjusted outcomes in the pre-CJR and post-CRJ periods and the adjusted DiD estimates comparing outcomes after the implementation of bundled payments and the announcement of participating MSAs. The CJR program was associated with a reduction of 2.6 (95% CI, 2.2 to 2.9) percentage points in discharge to institutional PAC for TM patients, a 6.1% relative reduction (*P* < .001), and a reduction of 1.5 (95% CI, 1.0 to 2.0) percentage points among MA patients, a 3.8% relative reduction (*P* < .001). In addition, the CJR program was associated with an increase of 2.1 (95% CI, 1.6 to 2.6) percentage points in discharge to home with home health care among TM patients (*P* < .001) but was not associated with a change in discharge to home with home health care among MA patients (−0.5 percentage points; 95% CI, −1.2 to 0.2 percentage points; *P* = .13). The CJR program was not associated with a change in discharge home without home health care among TM patients (0.2 percentage points; 95% CI, −0.2 to 0.7 percentage points; *P* = .34) but was associated with an increase of 1.8 (95% CI, 1.0 to 2.5) percentage points among MA patients (*P* < .001). The CJR program was also associated with significant reductions in discharge to SNFs and IRFs among both MA and TM enrollees (SNF: TM, −0.6 percentage points; 95% CI, −1.0 to −0.3 percentage points; *P* < .001; MA, −0.8 percentage points; 95% CI, −1.3 to −0.3 percentage points; *P* = .002; IRF: TM, −1.9 percentage points; 95% CI, −2.1 to −1.8 percentage points; *P* < .001; MA, −0.7 percentage points; 95% CI, −0.8 to −0.6 percentage points; *P* < .001).

**Table 2.** Changes in Discharge Location, Hospital Length of Stay, and Use of PAC Among TM and MA Patients in Bundled Payment and Control MSAs^a^

PAC Type	Control MSA		Bundled Payment MSA		Difference in Differences				
					Announcement		Implementation		Relative Change, %
	Pre-CJR	Post-CJR	Pre-CJR	Post-CJR	Coefficient (95% CI)	*P* Value	Coefficient (95% CI)	*P* Value	
**Discharge Location, %**									
Any institutional PAC									
TM	41.1	33.2	42.9	32.2	–2.0 (–2.4 to –1.6)	<.001	–2.6 (–2.9 to –2.2)	<.001	–6.1
MA	36.2	29.8	39.7	31.2	–1.4 (–1.9 to –0.9)	<.001	–1.5 (–2.0 to –1.0)	<.001	–3.8
Home with home health care^b^									
TM	33.6	34.8	35.9	38.7	1.1 (0.7 to 1.5)	<.001	2.1 (1.6 to 2.6)	<.001	5.8
MA	26.8	27.7	29.1	30.3	–0.7 (–1.2 to –0.1)	.02	–0.5 (–1.2 to 0.2)	.13	–1.7
Home without home health care^b^									
TM	25.1	31.8	21.0	28.8	0.1 (–0.2 to 0.5)	.42	0.2 (–0.2 to 0.7)	.34	1.0
MA	36.7	39.6	30.9	35.0	1.6 (1.1 to 2.2)	<.001	1.8 (1.0 to 2.5)	<.001	5.8
SNF									
TM	34.0	27.9	35.0	27.8	–0.0 (–0.4 to 0.3)	.90	–0.6 (–1.0 to –0.3)	<.001	–1.7
MA	34.4	28.3	37.2	30.0	–0.7 (–1.2 to –0.2)	.009	–0.8 (–1.3 to –0.3)	.002	–2.2
IRF									
TM	6.2	4.9	7.4	4.1	–2.0 (–2.2 to –1.8)	<.001	–1.9 (–2.1 to –1.8)	<.001	–25.7
MA	1.60	1.20	2.20	0.90	–0.7 (–0.8 to –0.5)	<.001	–0.7 (–0.8 to –0.6)	<.001	–31.8
**Time in PAC, d**									
Any institutional PAC									
TM	13.4	10.9	14.3	10.9	–0.6 (–0.7 to –0.4)	<.001	–0.8 (–0.9 to –0.7)	<.001	–5.6
MA	11.2	9.5	12	9.9	–0.4 (–0.6 to –0.2)	<.001	–0.3 (–0.5 to –0.2)	<.001	–2.5
Acute hospital									
TM	3.6	3.2	3.8	3.4	–0.02 (–0.05 to 0.0)	.14	–0.03 (–0.05 to 0.0)	.06	–0.8
MA	3.6	3.2	3.7	3.3	–0.04 (–0.08 to 0.0)	.08	–0.01 (–0.05 to 0.0)	.64	–0.3
SNF									
TM	8.7	7	9.6	6.9	–0.4 (–0.5 to 0.2)	<.001	–0.6 (–0.7 to 0.4)	<.001	–6.3
MA	7.4	6.1	8.1	6.5	–0.3 (–0.5 to –0.1)	<.001	–0.3 (–0.4 to –0.1)	.002	–3.7
IRF									
TM	0.8	0.6	0.9	0.6	–0.2 (–0.2 to –0.2)	<.001	–0.2 (–0.2 to –0.2)	<.001	–22.2
MA	0.2	0.2	0.3	0.1	–0.1 (–0.1 to –0.1)	<.001	–0.1 (–0.1 to –0.1)	<.001	–33.3
Home with home health care^b^									
TM	16.2	15	16.6	14.6	–0.3 (–0.5 to –0.2)	<.001	–0.4 (–0.6 to –0.2)	<.001	–2.4
MA	12.7	12.6	12.4	11.9	–0.2 (–0.4 to 0.0)	.06	–0.4 (–0.6 to –0.1)	.005	–3.2
No PAC^b^									
TM	60.5	64.1	59.2	64.5	0.7 (0.4 to 0.9)	<.001	1.1 (0.9 to 1.4)	<.001	1.9
MA	66.1	67.3	65.6	67.5	0.6 (0.3 to 0.9)	<.001	0.7 (0.3 to 1.0)	<.001	1.1
**Other Outcomes**									
Hospital length of stay, d									
TM	3	2.7	3.2	2.8	–0.01 (0.02 to 0.01)	.44	–0.02 (–0.03 to 0.02)	.045	–0.6
MA	3	2.7	3.1	2.8	0.01 (–0.01 to 0.03)	.45	0.01 (–0.01 to 0.03)	.37	–0.3
Cost per episode, $^b^									
TM	22 920.60	20 044.70	21 205.30	20 376.20	–639.7 (–797.6 to –481.8)	<.001	–767.4 (–920.5 to –614.4)	<.001	–3.6
MA	20 736.60	18 221.70	21 533.70	18 579.80	–439.5 (–661.3 to –217.7)	<.001	–348.0 (–560.6 to –135.5)	.001	–1.6

Abbreviations: CJR, Comprehensive Care for Joint Replacement; IRF, inpatient rehabilitation facility; MA, Medicare Advantage; MSA, metropolitan statistical area; PAC, postacute care; SNF, skilled nursing facility; TM, traditional Medicare.

^a^ All models are linear probability models at the level of episode of care, adjusted for age, sex, race/ethnicity, dual eligibility, diagnosis related group, and hospital fixed effects. Models were weighted by the probability of selection in treatment MSA. Numbers before and after CJR are unadjusted. The first difference-in-differences estimate includes quarter 1 of 2013 to quarter 2 of 2015 as the pre-CJR period and quarter 3 of 2015 to quarter 3 of 2017 as the post-CJR period, corresponding to the announcement of CJR. The second difference-in-differences estimate includes quarter 1 of 2013 to quarter 1 of 2016 as the pre-CJR period and quarter 2 of 2016 to quarter 3 of 2017 as the post-CJR period, corresponding to the implementation of bundling. Data for discharge to home health and days at home were not available in 2017.

^b^ Outcome does not include 2017 data as a result of limited home health care information. The percentage difference is calculated from the second difference-in-differences estimates. All variables are calculated from the residential history file.

The CJR program was associated with significant reductions in the number of days spent in PAC for both MA and TM patients. The CJR program was associated with a decrease of 0.8 (95% CI, 0.7-0.9) days in any institutional PAC setting among TM patients (*P* < .001) and a decrease of 0.3 (95% CI, 0.2-0.5) days among MA patients (*P* < .001), a 5.6% and 2.5% relative reduction, respectively. The largest relative changes in PAC were 22.2% and 33.3% relative reductions in IRF days for TM and MA, respectively (TM, 0.2 days; 95% CI, 0.2-0.2 days; *P* < .001; MA, 0.1 days; 95% CI, 0.1-0.1 days; *P* < .001).

In Table 2, we present changes in expenditures and initial hospital length of stay. Bundled payments were also associated with a reduction in expenditures (actual among TM patients and extrapolated among MA patients) of about $767.4 (95% CI, $614.4-$920.5) among TM patients (*P* < .001) and $348.0 (95% CI, $135.5-$560.6) among MA patients (*P* = .001). There were minimal changes in initial length of stay in the hospital. All models met the parallel trends assumption (eTable 3 in the Supplement). Across all outcomes, results were similar when using a post-CJR period defined by the announcement of the CJR program (Table 2).

**Figure 2.**
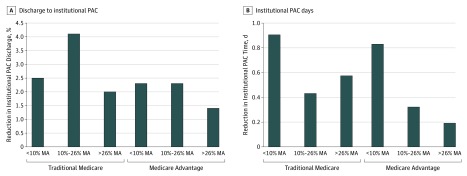
Reductions in Discharge to Institutional Postacute Care (PAC) and Institutional Postacute Days After Bundled Payments, by the Hospital’s Proportion of Medicare Advantage (MA) Patients Undergoing Joint Replacement Medicare Advantage penetration is calculated as a percentage of all joint replacement admissions for MA patients. Tertile 1 is less than 10% MA patients; tertile 2 is 10% to 26%; tertile 3, greater than 26%. Institutional PAC includes days spent in skilled nursing facilities or inpatient rehabilitation facility.

Figure 2 shows the DiD estimates for discharge to institutional PAC settings and total institutional PAC days among MA and TM patients, stratified by the hospital’s proportion of MA patients. As MA concentration increased, the reductions in institutional days became smaller among both MA and TM patients. In hospitals with low concentrations of MA patients, time spent in institutional PAC settings decreased by 0.9 (95% CI, 0.6 to 1.2) days among TM patients (*P* < .001) and 0.8 (95% CI, −0.8 to 2.5) days among MA patients (*P* = .67); in hospitals with high MA concentrations, time spent in institutional PAC settings decreased by 0.6 (95% CI, 0.4 to 0.8) days for TM patients (*P* < .001) and 0.2 (95% CI, 0.1 to 0.3) days for MA patients (*P* < .001). The hospitals with the highest proportion of MA patients also had the lowest effect size for discharge to institutional PAC (a 2.5 [95% CI, 1.3 to 3.7] percentage point reduction among TM patients [*P* < .001] and a 2.3 [95% CI, 1.5 to 3.5] percentage point reduction among MA patients [*P* < .001] in hospitals with low MA concentrations and a 2.0 [95% CI, 1.5 to 2.5] percentage point reduction among TM patients [*P* < .001] and a 1.4 [95% CI, 1.0 to 1.9] percentage point reduction among MA patients [*P* < .001] in hospitals with high MA concentrations).

In the triple DiD models, the CJR program led to a significantly larger reduction among TM patients compared with MA patients in discharges to institutional PAC settings (1.3 percentage points; 95% CI, 0.8 to 1.9 percentage points; *P* < .001), discharges home without home health care (1.2 percentage points; 95% CI, 0.3 to 2.1 percentage points; *P* = .004), discharges to IRFs (1.6 percentage points; 95% CI, 1.3 to 1.8 percentage points; *P* < .001), institutional PAC days (0.5 days; 95% CI, 0.3 to 0.7 days; *P* < .001), IRF days (0.2 days; 95% CI, 0.1 to 0.2 days; *P* < .001), and costs ($386.90; 95% CI, $125.5 to $648.4; *P* < .001). The CJR program led to larger reductions among MA patients compared with TM patients in discharge to home with home health care (−2.6 percentage points; 95% CI, −3.4 to −1.7 percentage points; *P* < .001) and days without PAC (−0.5 days; 95% CI, −1.0 to −0.1 days; *P* = .02) (eTable 2 in the Supplement).

In sensitivity analyses, the trends were largely similar when using alternative pre-CJR and post-CJR specifications, when excluding hospital fixed effects, when using hospital random effects, and when removing probability weighting (eTable 4 in the Supplement). There was no significant association between the CJR program and the number of joint replacements performed in the treatment vs control MSAs (eTable 5 in the Supplement). Full primary model output is included in eTable 6 in the Supplement.

## Discussion

Mandatory bundled payments for TM patients receiving joint replacement surgical procedures were associated with significant reductions in institutional PAC use and associated spending for MA patients. The associations were larger in hospitals with lower proportions of MA patients. Our analysis adds to the literature on spillovers (ie, the unintended consequences a policy targeting one population has on another population) between MA and TM health care utilization and found a spillover to MA patients from a change in TM policy. The association of bundled payments with PAC use and cost among MA patients may occur if hospitals changed their care management practices for all patients receiving joint replacement, regardless of insurance status. Prior work has found that increases in MA penetration in an area may affect the use of services among TM patients.^4,9,10,11,12^ We found that the potential for spillovers may be bidirectional; in other words, alternative payment models in TM may affect care delivery among patients enrolled in MA. Furthermore, we found that hospitals with lower concentrations of MA admissions had larger reductions in PAC use, which may be because these hospitals had greater financial risk. The DiD estimates were generally smaller for MA than TM, which may be in part because patients enrolled in MA already have lower rates of PAC use.^6,13^

A prior study^5^ has found an association of the CJR program with MA patients. Our study found similar results and improves on the past work in 5 key ways. First, we used 18 months of follow-up data rather than 6 months when evaluating the success of the program. Second, the number of days spent in each setting in the 90 days after surgery is a more informative measure of PAC use and spending than a measure of hospital discharge location alone. Third, this study found that the MedPAR discharge code variable was inaccurate (eTable 1 in the Supplement). In particular, the MedPAR discharge code may overestimate the number of patients actually receiving PAC, particularly for home health services. Our use of assessment data may present a more complete picture of the PAC services that patients actually receive. Fourth, we evaluated the potential consequences of the policy before and after its announcement in addition to before and after its implementation. Fifth, we provided more detail on between-hospital heterogeneity in outcomes and found differences in the associations of the intervention with outcomes between hospitals that had different concentrations of MA patients.

Our analysis of trends illustrated that, since 2013, PAC use has been steadily decreasing in terms of both initial discharge to PAC settings and the duration of PAC use in the 90 days after joint replacement surgery in control and treatment hospitals. Our study suggests that the CJR program may have accelerated this reduction. We also found that the association of the intervention with outcomes appeared to begin after the announcement of the CJR bundle program, ie, before the implementation of bundled payments. Once hospitals knew they would be subject to bundling, they may have changed their care processes immediately. Thus, evaluations of the CJR program that do not also consider the changes between the announcement and the start of financial risk may underestimate the overall consequences of the program. The decrease over the same period in control hospitals may be a continuation of a secular trend toward lower PAC use.

We found that savings from the program may be associated with reductions in all types of PAC use as well as the substitution of home health care for institutional PAC settings. The shift to home health care appeared more pronounced among TM patients, while, by the end of the study period, MA patients were more likely to be discharged home without home health care. However, both TM and MA patients received fewer days of home health care after the introduction of the CJR program. We cannot assess whether these patients received other types of outpatient treatments in place of certified Medicare Home Health services, although studies of TM patients^2^ found little change in outpatient encounters and spending following the CJR program.

Our findings have important policy implications and open new avenues for study as CMS implements other alternative payment models that may affect care outside the TM program. Under the current CJR model, hospitals are the only entities with higher levels of risk, yet MA plans may passively benefit from reductions in spending and use of PAC among patients treated in participating hospitals. The Centers for Medicare & Medicaid Services could therefore consider partnering with MA plans in bundled payment initiatives, particularly because the association of the CJR program with PAC use were attenuated in hospitals caring for larger proportions of MA patients. Additionally, evaluations and financial scoring of the CJR program that do not consider the association of the program with MA patients may substantially understate the full extent of the consequences of the CJR bundled payment program.

Our study also suggested investigation of the consequences of the reduced intensity of institutional PAC use and the shift to less expensive forms of PAC on patient-centered outcomes, such as physical functioning and health-related quality of life. While past work has compared differences in physical functioning and mortality between patients discharged to home with home health care compared with those discharged to SNFs, the focus was not on patients undergoing joint replacement.^14^ Our study found that this practice was more common among MA patients and increased following the implementation of the CJR program. A randomized clinical trial of home health care vs inpatient rehabilitation^15^ following knee arthroplasty failed to detect differences in outcomes, but randomized trial studies among patients undergoing joint replacement are lacking.^14^ Future work is needed to ensure that bundled payments are not associated with adverse unintended consequences because of lower use of effective PAC and rehabilitative services.

### Limitations

Our study has limitations. First, our analysis may have missed MA patients who received joint replacements from hospitals that do not report to MedPAR; however, it is unlikely that reporting would differ in the randomized treatment and control MSAs, and our sensitivity analysis found that 90% of joint replacements in these MSAs were at hospitals with mandatory reporting of MA records. Second, we did not have detailed data on actual MA spending for our calculation of expenditures. We therefore estimated spending if reimbursements were similar to those in TM. While this calculation of costs is not ideal, national data on spending in the MA program are not available. Third, we used an intent-to-treat approach in our analysis; however, not all MSAs randomized to treatment actually participated in the bundles. As a result, our findings may underestimate the association of the intervention with outcomes. Fourth, when we calculated the percentage of MA patients in a hospital, we only had data on Medicare beneficiaries, so we could not account for commercial or non–dually eligible Medicaid enrollees in that analysis.

## Conclusions

In conclusion, we found that the CJR program was associated with spillovers from the TM population to those enrolled in MA in the form of lower PAC use. The Centers for Medicare & Medicaid Services bundled payment models may catalyze changes in care delivery that extend beyond the TM program.

## Notes

Supplement. **eFigure. **Trends in Outcomes for Medicare Advantage and Traditional Medicare Enrollees in Treatment and Control MSAs **eTable 1. **Concordance Between MedPAR and RHF Discharge Measures **eTable 2. **Triple Difference Results **eTable 3. **Tests of Parallel Trends **eTable 4. **Sensitivity Checks **eTable 5. **Change in Total Joint Replacements Conducted at Hospitals **eTable 6. **Full Regression Output for Primary Models
