# Supplementary material for: Association of Mandatory Bundled Payments for Joint Replacement With Use of Postacute Care Among Medicare Advantage Enrollees
Source: JAMA Netw Open. 2019 Dec 27;2(12):e1918535. doi: 10.1001/jamanetworkopen.2019.18535 (PMC6991238; doi:10.1001/jamanetworkopen.2019.18535)
Supplement: Supplement. — eFigure. Trends in Outcomes for Medicare Advantage and Traditional Medicare Enrollees in Treatment and Control MSAs eTable 1. Concordance Between MedPAR and RHF Discharge Measures eTable 2. Triple Difference Results eTable 3. Tests of Parallel Trends eTable 4. Sensitivity Checks eTable 5. Change in Total Joint Replacements Conducted at Hospitals eTable 6. Full Regression Output for Primary Models [file jamanetwopen-2-e1918535-s001.pdf]

Supplementary Online Content

Meyers DJ, Kosar CM, Rahman M, Mor V, Trivedi AN. Association of mandatory bundled payments for joint replacement with use of postacute care among Medicare Advantage enrollees. *JAMA Netw Open*. 2019;2(12):e1918535. doi:10.1001/jamanetworkopen.2019.18535

eFigure. Trends in Outcomes for Medicare Advantage and Traditional Medicare Enrollees in Treatment and Control MSAs

eTable 1. Concordance Between MedPAR and RHF Discharge Measures

eTable 2. Triple Difference Results

eTable 3. Tests of Parallel Trends

eTable 4. Sensitivity Checks

eTable 5. Change in Total Joint Replacements Conducted at Hospitals

eTable 6. Full Regression Output for Primary Models

This supplementary material has been provided by the authors to give readers additional information about their work.

© 2019 Meyers DJ et al. *JAMA Network Open.*

eFigure. Trends in Outcomes for Medicare Advantage and Traditional Medicare Enrollees in Treatment and Control MSAs

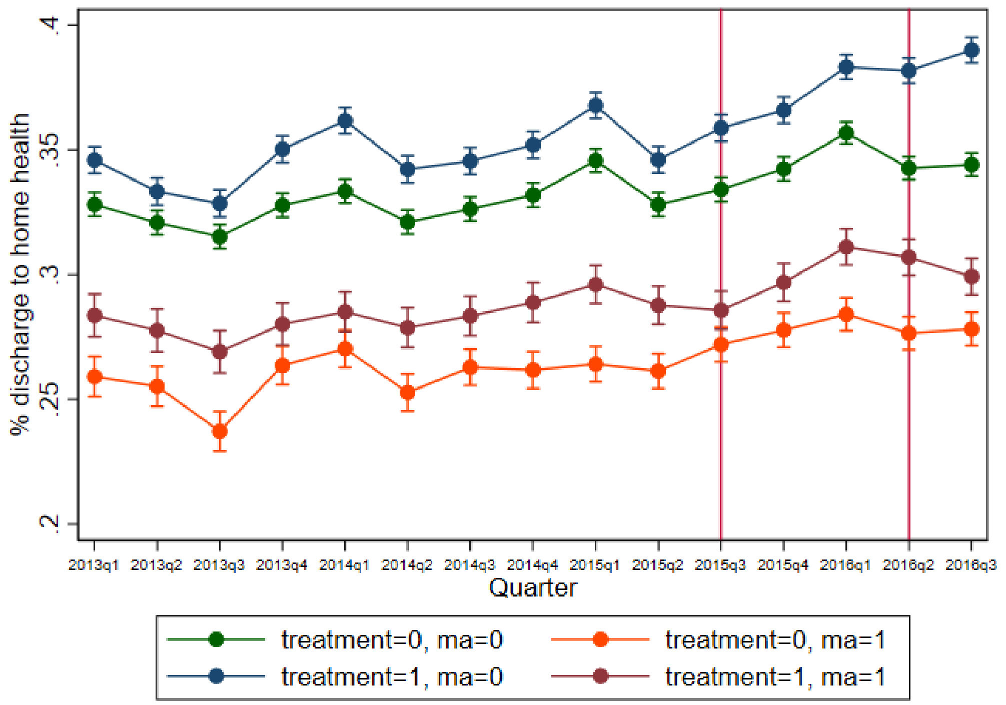

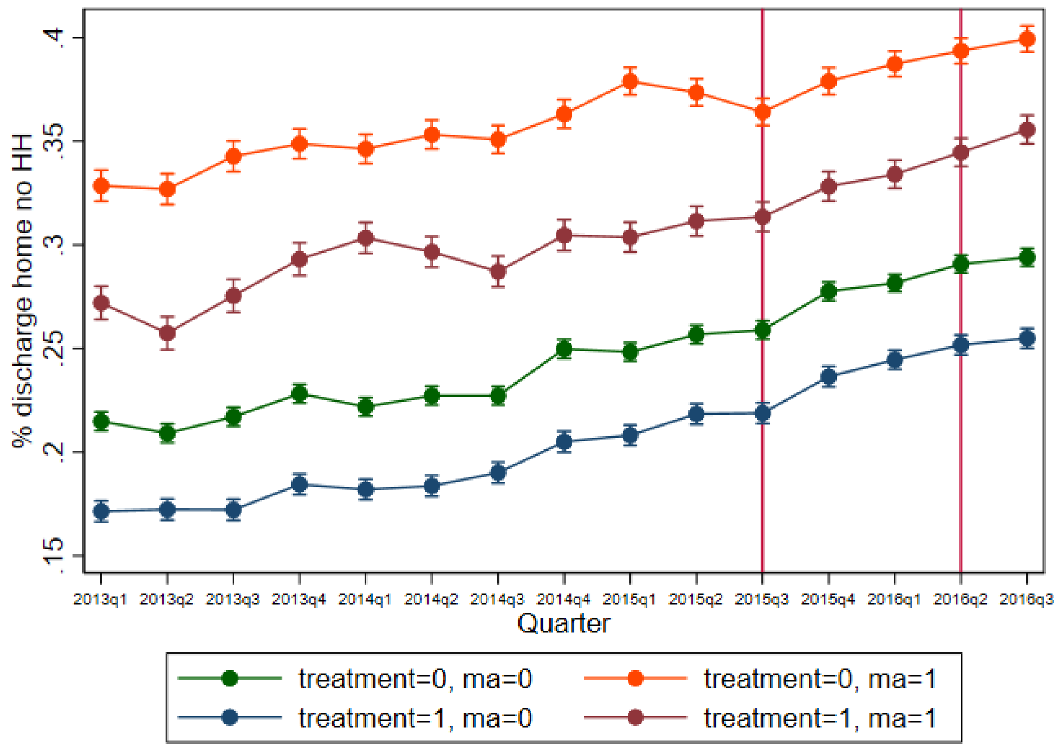

© 2019 Meyers DJ et al. *JAMA Network Open.*

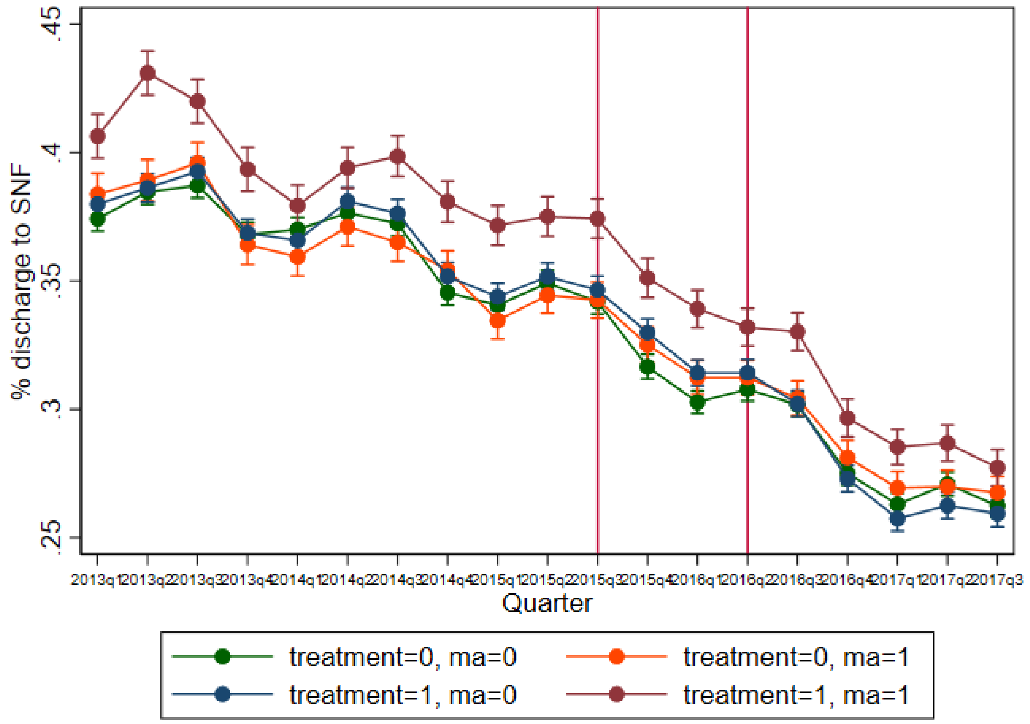

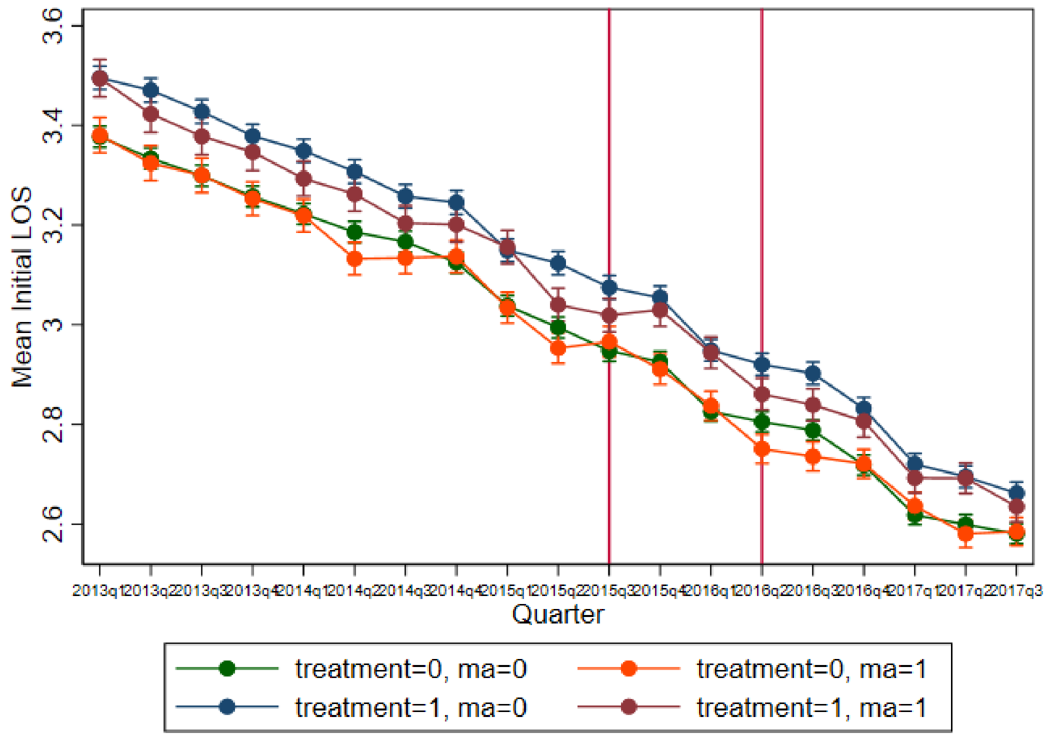

© 2019 Meyers DJ et al. *JAMA Network Open.*

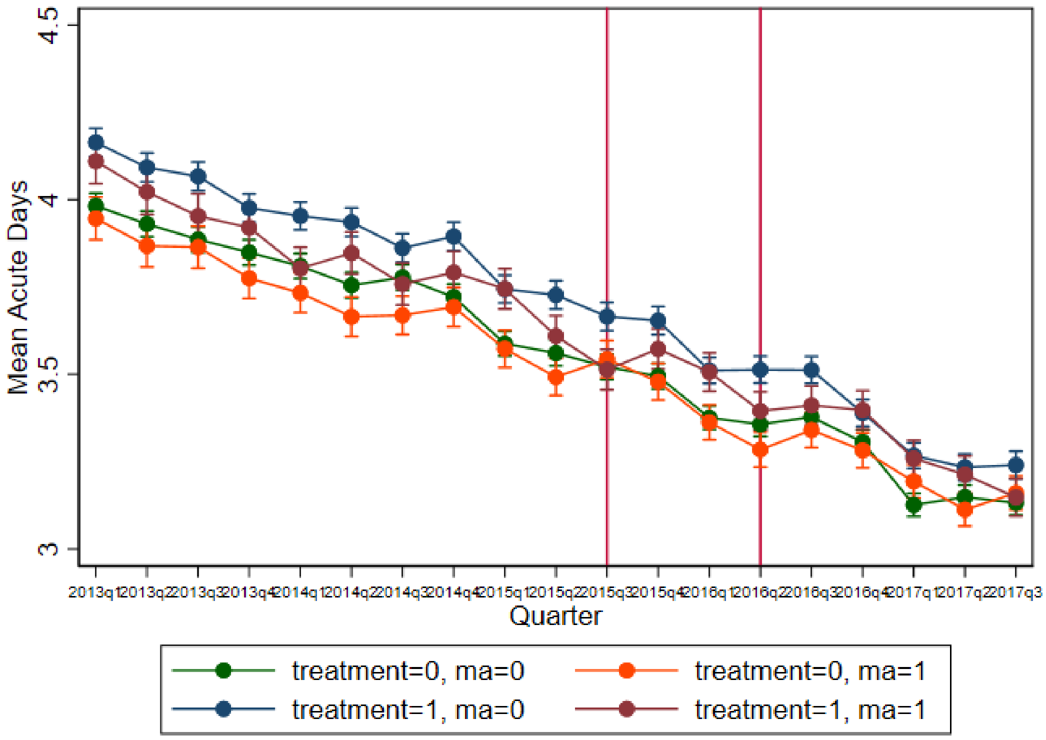

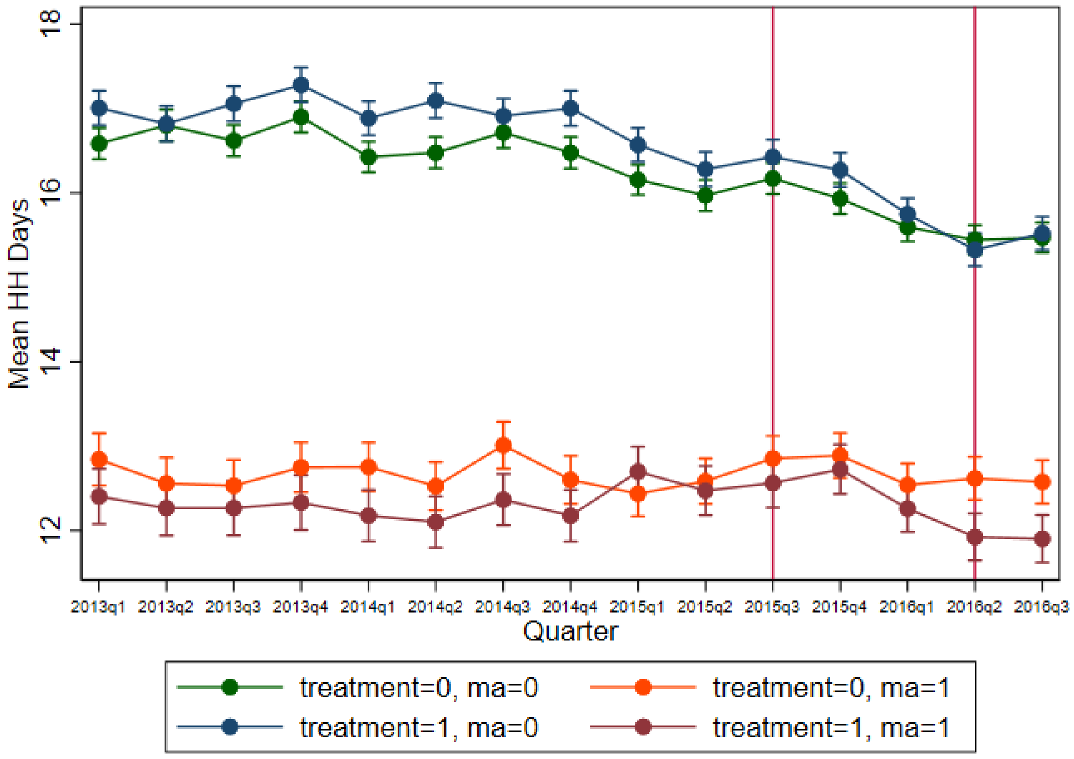

© 2019 Meyers DJ et al. *JAMA Network Open.*

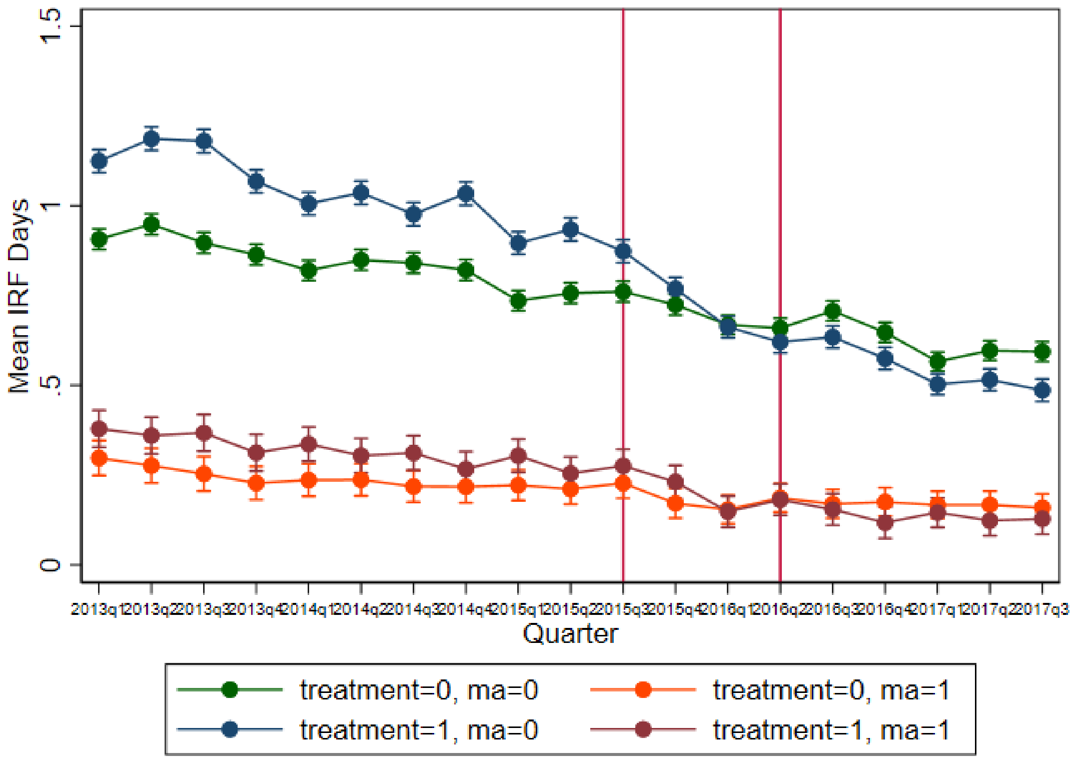

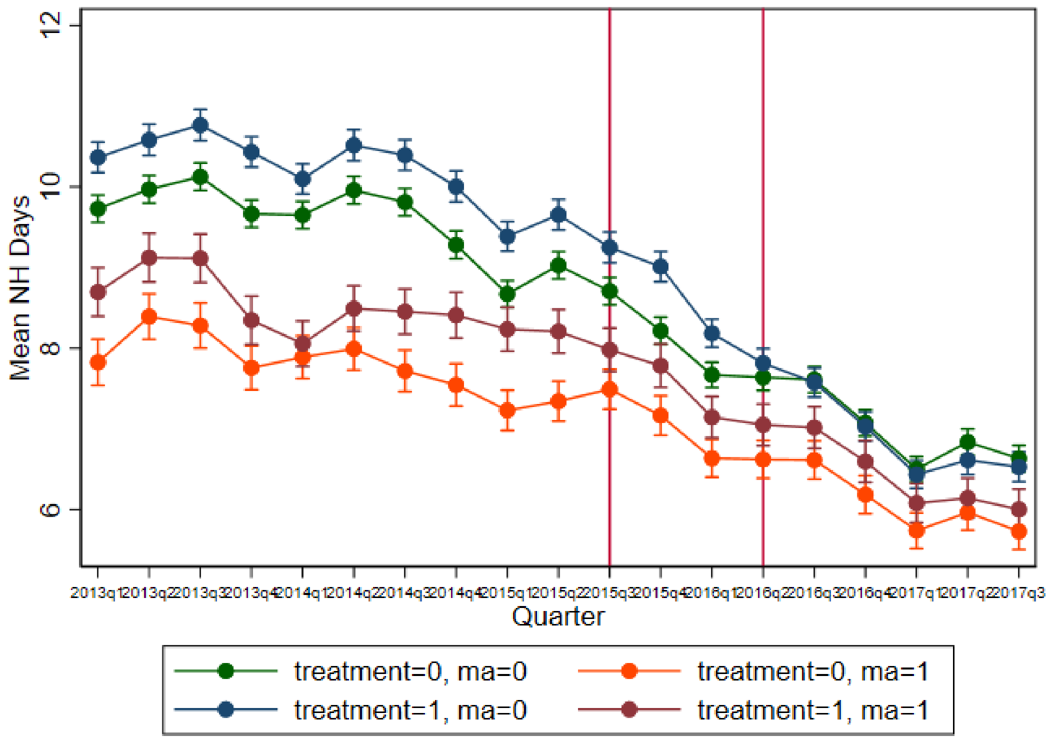

© 2019 Meyers DJ et al. *JAMA Network Open.*

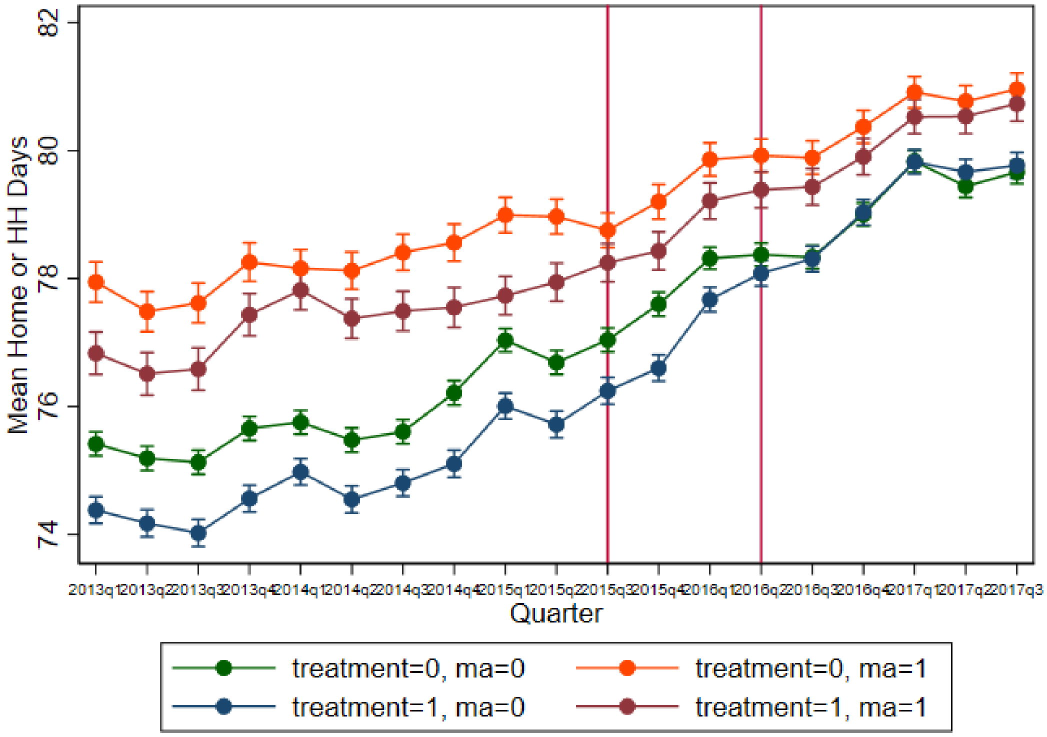

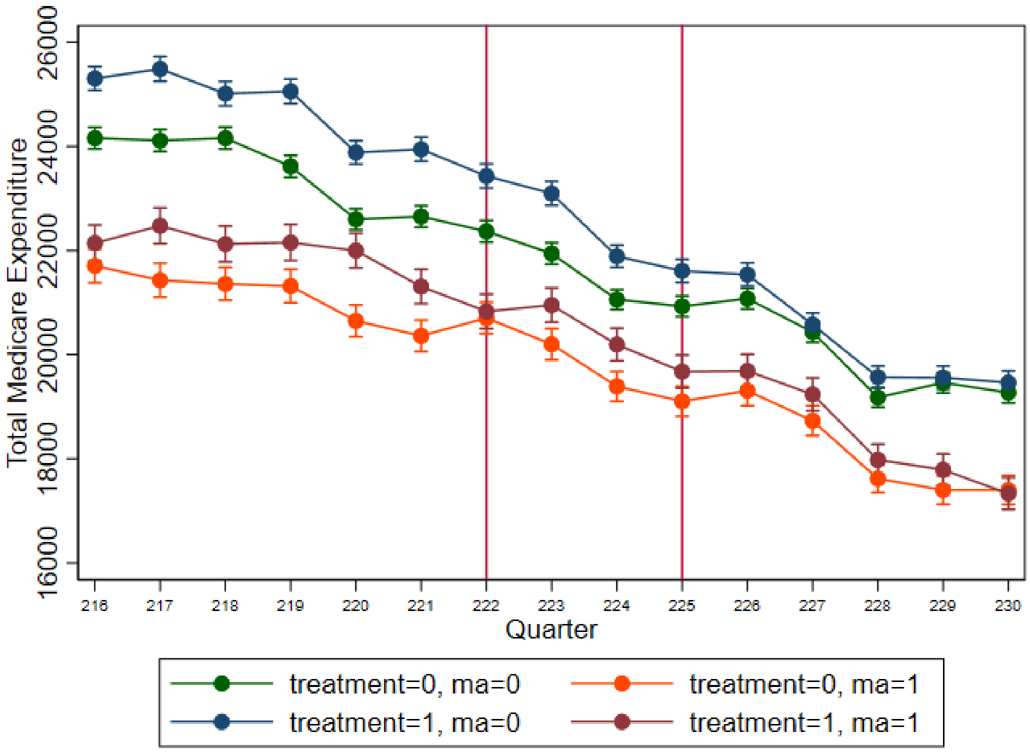

Notes: In all plots, the first line represents when the CJR was announced. The second line represents when financial penalties for the CJR began. Plots are otherwise unadjusted quarterly trends.

© 2019 Meyers DJ et al. *JAMA Network Open.*

eTable 1. Concordance Between MedPAR and RHF Discharge Measures

	TM		MA	
	Concordance	% Captured by MedPAR	Concordance	% Captured by MedPAR
SNF	95.4%	89.3%	95.0%	91.9%
IRF	93.9%	91.5%	95.5%	87.2%
HH	92.4%	87.2%	82.6%	90.5%

Notes: Table displays the concordance statistic between the MedPAR discharge code and RHF based discharge for skilled nursing facility, inpatient rehabilitation facility, and home health. With the RHF as the gold standard, the table also displays the % of those discharged based on the RHF that were also successfully captured by the MedPAR.

© 2019 Meyers DJ et al. *JAMA Network Open.*

eTable 2. Triple Difference Results

	Triple Difference Coefficient	95%CI
Discharge Location		
Institutional PAC	1.3**	(0.8 , 1.9)
Home Health	-2.6**	(-3.4 ,-1.7)
Home without Home Health	1.2**	(0.3 , 2.1)
IRF	1.6**	(1.3 , 1.8)
SNF	-0.2	(-0.8 ,0.3)
Number of Days in Follow-up		
Any Institutional PAC	0.5**	(0.3 , 0.7)
Home Health	0.1	(-.2 , 0.4)
Acute	0.02	(-0.3 ,0.6)
SNF	0.3**	(0.1 , 0.5)
IRF	0.2**	(0.1 , 0.2)
No PAC	-0.5*	(-1.0 , -0.08)
Other Outcomes		
Initial Length of Stay	0.03	(0.005 , 0.06)
Total Costs	386.9*	(125.5 , 648.4)

Notes: Coefficients in this table are from triple difference in difference models. These coefficients can be interpreted as the difference in the differences between the control and treated groups, for MA as compared to TM.

© 2019 Meyers DJ et al. *JAMA Network Open.*

eTable 3. Tests of Parallel Trends

Outcome		p-value
Discharge Location		
Any Inpatient	TM	0.269
	MA	0.320
Home Health	TM	0.319
	MA	0.441
Home Without Home Health	TM	0.771
	MA	0.099
SNF	TM	0.817
	MA	0.362
Initial Length of Stay	TM	0.567
	MA	0.637
Number of Days in 90 Follow-up		
Home Health	TM	0.116
	MA	0.466
Acute Hospital	TM	0.998
	MA	0.503
SNF	TM	0.340
	MA	0.711
IRF	TM	0.067
	MA	0.573
Any Institutional PAC	TM	0.763
	MA	0.395
No PAC	TM	0.174
	MA	0.932
Cost	TM	.293
	MA	.936

Notes: all p-values are taken from models estimating the interaction of quarter and treatment group in the pre-period to test if the parallel trends assumption is met. P>0.05 is indicative that there does not appear to be significant differences in parallel trends.

© 2019 Meyers DJ et al. *JAMA Network Open.*

eTable 4. Sensitivity Checks

		Any Institutional Discharge		Number of Institutional Days	
		Difference in Difference	95% CI	Difference in Difference	95% CI
No Hospital FE	TM	-2.4**	(-2.9 , -2.0)	-2.1**	(-2.3 , -1.9)
	MA	-1.7**	(-2.3 , -0.4)	-1.0**	(-1.2 , -0.8)
Excluding 2015	TM	-2.3**	(-2.8, -1.9)	-2.1**	(-2.3 , -1.8)
	MA	-1.5**	(-2.2 , -1.0)	-0.9**	(-1.2 , -0.7)
Without Probability Weights	TM	-2.8**	(-3.2 , -2.4)	-2.3**	(-2.5 , -2.1)
	MA	-1.7**	(-2.1 , -1.2)	-0.9**	(-1.1 , -0.7)
Hospital Random Effects	TM	-2.9**	(-3.1, -2.4)	-2.3**	(-2.5 , -2.1)
	MA	-1.7**	(-2.2, -1.1)	-0.9**	(-1.1 , -0.7)

Notes**:** Table displays difference in difference coefficients using the primary model specifications under different sensitivity specifications. The two primary outcomes of any inpatient discharge and number of inpatient days are displayed. Coefficients represent percentage point difference in differences pre and post the intervention. * denotes p<0.05, ** denotes p<0.001

© 2019 Meyers DJ et al. *JAMA Network Open.*

eTable 5. Change in Total Joint Replacements Conducted at Hospitals

	Enrollee Type	Control		Bundled Payment		Difference in Difference		
		Pre-CJR	Post-CJR	Pre-CJR	Post-CJR	Coef	95%CI	p-value
Total Joint Replacements	TM	409.6	308.3	393.3	292.7	-0.2	(-3.6, 3.1)	0.9
	MA	190.1	160.6	189.7	154.4	-0.5	(-2.3, 1.3)	0.569

Notes: DiD estimates come from models detailed further in the text. Values are hospital level quarterly counts of joint replacements.

© 2019 Meyers DJ et al. *JAMA Network Open.*

eTable 6. Full Regression Output for Primary Models

	Discharge Institutional PAC		Discharge HH		Discharge Home no HH		Discharge to SNF		Length of Stay		Home Health Days		Acute Days		Nursing Home Days		IRF Days		Total Institutional PAC Days		No PAC Days		Discharge to IRF		Expenditure	
VARIABLES	MA	TM	MA	TM	MA	TM	MA	TM	MA	TM	MA	TM	MA	TM	MA	TM	MA	TM	MA	TM	MA	TM	MA	TM	MA	TM
Post	-0.123**	-0.106**	0.014**	0.019**	0.065**	0.046**	-0.100**	-0.095**	-0.607**	-0.667**	-0.858**	-0.179	-0.615**	-0.623**	-2.738**	-2.085**	-0.230**	-0.112**	-3.604**	-2.821**	3.269**	1.909**	-0.024**	-0.011**	-4,479.163**	-4,507.103**
	(-0.129 - -0.118)	(-0.113 - -0.099)	(0.008 - 0.019)	(0.011 - 0.026)	(0.060 - 0.069)	(0.039 - 0.054)	(-0.105 - -0.095)	(-0.102 - -0.088)	(-0.629 - -0.586)	(-0.701 - -0.634)	(-1.061 - -0.656)	(-0.457 - 0.098)	(-0.653 - -0.577)	(-0.678 - -0.567)	(-2.919 - -2.558)	(-2.313 - -1.856)	(-0.263 - -0.197)	(-0.141 - -0.084)	(-3.804 - -3.404)	(-3.076 - -2.566)	(2.986 - 3.552)	(1.511 - 2.306)	(-0.026 - -0.021)	(-0.013 - -0.009)	(-4,695.150 - -4,263.175)	(-4,807.837 - -4,206.369)
Post * Treatment	-0.026**	-0.015**	0.021**	-0.005	0.002	0.018**	-0.006**	-0.008**	-0.015*	0.010	-0.425**	-0.371**	-0.026	-0.009	-0.563**	-0.251**	-0.208**	-0.073**	-0.801**	-0.331**	1.136**	0.675**	-0.019**	-0.007**	-767.423**	-348.041**
	(-0.029 - -0.022)	(-0.020 - -0.010)	(0.016 - 0.026)	(-0.012 - 0.002)	(-0.002 - 0.007)	(0.010 - 0.025)	(-0.010 - -0.003)	(-0.013 - -0.003)	(-0.030 - -0.000)	(-0.012 - 0.033)	(-0.622 - -0.228)	(-0.631 - -0.110)	(-0.053 - 0.001)	(-0.049 - 0.030)	(-0.690 - -0.437)	(-0.409 - -0.093)	(-0.231 - -0.185)	(-0.092 - -0.055)	(-0.941 - -0.660)	(-0.508 - -0.155)	(0.859 - 1.413)	(0.301 - 1.049)	(-0.021 - -0.018)	(-0.008 - -0.006)	(-920.497 - -614.350)	(-560.552 - -135.530)
Race =black (ref=white)	0.018**	0.022**	-0.006**	-0.006*	-0.014**	-0.021**	0.016**	0.026**	-0.013	-0.018	2.236**	1.762**	-0.040*	-0.088**	-1.344**	-0.662**	0.016	-0.032**	-1.351**	-0.770**	-0.979**	-1.121**	0.004**	-0.002**	-483.389**	-511.937**
	(0.014 - 0.022)	(0.017 - 0.027)	(-0.010 - -0.001)	(-0.012 - -0.001)	(-0.018 - -0.011)	(-0.026 - -0.016)	(0.012 - 0.019)	(0.021 - 0.031)	(-0.031 - 0.005)	(-0.041 - 0.005)	(2.044 - 2.428)	(1.537 - 1.987)	(-0.073 - -0.007)	(-0.129 - -0.047)	(-1.488 - -1.201)	(-0.816 - -0.509)	(-0.011 - 0.043)	(-0.051 - -0.012)	(-1.511 - -1.191)	(-0.942 - -0.598)	(-1.231 - -0.727)	(-1.427 - -0.815)	(0.002 - 0.006)	(-0.004 - -0.001)	(-664.728 - -302.050)	(-725.372 - -298.502)
Race =hispanic	0.032**	-0.014*	-0.004	0.004	-0.029**	0.004	0.025**	-0.013*	-0.004	-0.058*	0.983**	-0.411	-0.232**	-0.192**	-1.765**	-1.340**	0.073*	0.001	-1.979**	-1.521**	0.858**	1.985**	0.010**	0.000	-1,571.767**	-1,436.644**
	(0.023 - 0.042)	(-0.027 - -0.002)	(-0.015 - 0.007)	(-0.009 - 0.018)	(-0.038 - -0.021)	(-0.011 - 0.019)	(0.015 - 0.034)	(-0.026 - -0.001)	(-0.052 - 0.044)	(-0.114 - -0.002)	(0.511 - 1.456)	(-0.925 - 0.104)	(-0.313 - -0.151)	(-0.293 - -0.091)	(-2.148 - -1.383)	(-1.762 - -0.918)	(0.006 - 0.139)	(-0.041 - 0.042)	(-2.393 - -1.565)	(-1.985 - -1.057)	(0.229 - 1.487)	(1.227 - 2.743)	(0.005 - 0.016)	(-0.003 - 0.003)	(-2,014.287 - -1,129.248)	(-1,978.729 - -894.559)

© 2019 Meyers DJ et al. *JAMA Network Open.*

Race =asian	-0.070**	-0.057**	0.044**	0.019**	0.027**	0.030**	-0.058**	-0.054**	-0.249**	-0.232**	2.127**	-0.026	-0.511**	-0.415**	-4.566**	-2.915**	-0.177**	-0.017	-5.273**	-3.333**	2.937**	3.288**	-0.010**	-0.001	-4,280.083**	-3,216.396**
	(-0.079 - -0.062)	(-0.066 - -0.048)	(0.034 - 0.054)	(0.010 - 0.029)	(0.019 - 0.034)	(0.019 - 0.040)	(-0.067 - -0.049)	(-0.063 - -0.045)	(-0.292 - -0.205)	(-0.275 - -0.189)	(1.641 - 2.613)	(-0.445 - 0.393)	(-0.586 - -0.436)	(-0.493 - -0.338)	(-4.918 - -4.214)	(-3.204 - -2.626)	(-0.242 - -0.111)	(-0.063 - 0.029)	(-5.657 - -4.890)	(-3.655 - -3.011)	(2.330 - 3.543)	(2.721 - 3.854)	(-0.015 - -0.005)	(-0.005 - 0.003)	(-4,691.369 - -3,868.797)	(-3,615.546 - -2,817.246)
Race =na/ai	-0.031**	-0.016	-0.004	-0.005	0.041**	0.023	-0.029**	-0.015	0.008	0.018	0.427	-0.483	0.027	0.039	-1.425**	-0.846	0.023	-0.029	-1.319**	-0.811	0.917	1.083	0.000	-0.005	-452.104	-324.712
	(-0.045 - -0.016)	(-0.044 - 0.011)	(-0.021 - 0.013)	(-0.035 - 0.024)	(0.024 - 0.057)	(-0.011 - 0.056)	(-0.043 - -0.015)	(-0.043 - 0.012)	(-0.058 - 0.073)	(-0.139 - 0.174)	(-0.382 - 1.236)	(-1.657 - 0.690)	(-0.095 - 0.149)	(-0.239 - 0.317)	(-1.980 - -0.870)	(-1.768 - 0.077)	(-0.073 - 0.120)	(-0.146 - 0.088)	(-1.948 - -0.690)	(-1.848 - 0.227)	(-0.111 - 1.945)	(-0.630 - 2.796)	(-0.007 - 0.008)	(-0.012 - 0.003)	(-1,142.315 - 238.107)	(-1,723.435 - 1,074.011)
Race =other/unknown	-0.089**	-0.070**	0.045**	0.021**	0.046**	0.051**	-0.077**	-0.067**	-0.327**	-0.242**	-1.051**	-1.091**	-0.526**	-0.423**	-3.074**	-2.330**	-0.177**	-0.032*	-3.809**	-2.785**	4.853**	3.941**	-0.010**	-0.002	-4,061.075**	-3,102.750**
	(-0.094 - -0.083)	(-0.077 - -0.062)	(0.038 - 0.052)	(0.011 - 0.030)	(0.040 - 0.052)	(0.040 - 0.061)	(-0.082 - -0.072)	(-0.075 - -0.060)	(-0.348 - -0.306)	(-0.279 - -0.206)	(-1.298 - -0.804)	(-1.411 - -0.771)	(-0.562 - -0.489)	(-0.481 - -0.366)	(-3.226 - -2.921)	(-2.526 - -2.134)	(-0.207 - -0.147)	(-0.057 - -0.006)	(-3.979 - -3.638)	(-3.007 - -2.564)	(4.534 - 5.172)	(3.504 - 4.377)	(-0.012 - -0.007)	(-0.004 - 0.000)	(-4,262.477 - -3,859.673)	(-3,397.452 - -2,808.047)
Female (ref= male)	0.123**	0.100**	-0.053**	-0.030**	-0.075**	-0.077**	0.111**	0.099**	0.163**	0.121**	2.644**	2.176**	0.070**	0.009	2.553**	1.769**	0.112**	-0.007	2.719**	1.764**	-5.600**	-4.156**	0.012**	0.001	2,003.376**	1,142.793**
	(0.121 - 0.125)	(0.097 - 0.103)	(-0.055 - -0.051)	(-0.033 - -0.027)	(-0.076 - -0.073)	(-0.080 - -0.074)	(0.110 - 0.113)	(0.097 - 0.102)	(0.155 - 0.171)	(0.109 - 0.134)	(2.564 - 2.723)	(2.068 - 2.283)	(0.056 - 0.084)	(-0.012 - 0.030)	(2.490 - 2.616)	(1.689 - 1.848)	(0.100 - 0.124)	(-0.017 - 0.003)	(2.649 - 2.790)	(1.674 - 1.854)	(-5.712 - -5.488)	(-4.311 - -4.001)	(0.011 - 0.013)	(-0.000 - 0.001)	(1,923.873 - 2,082.879)	(1,030.392 - 1,255.195)
Dual (ref= non-dual)	0.138**	0.118**	-0.055**	-0.036**	-0.078**	-0.079**	0.133**	0.114**	0.492**	0.407**	4.009**	2.343**	0.918**	0.664**	9.145**	6.609**	0.028*	0.012	10.155**	7.258**	-14.011**	-9.856**	0.001	0.001*	8,714.023**	6,215.144**
	(0.134 - 0.141)	(0.113 - 0.122)	(-0.059 - -0.052)	(-0.040 - -0.031)	(-0.081 - -0.076)	(-0.084 - -0.075)	(0.130 - 0.136)	(0.110 - 0.119)	(0.476 - 0.509)	(0.385 - 0.429)	(3.839 - 4.178)	(2.142 - 2.543)	(0.888 - 0.949)	(0.626 - 0.703)	(8.977 - 9.313)	(6.420 - 6.798)	(0.006 - 0.050)	(-0.005 - 0.029)	(9.977 - 10.334)	(7.056 - 7.460)	(-14.240 - -13.782)	(-10.151 - -9.561)	(-0.001 - 0.003)	(0.000 - 0.003)	(8,545.529 - 8,882.518)	(6,008.287 - 6,422.000)

© 2019 Meyers DJ et al. *JAMA Network Open.*

Constant	0.352**	0.315**	0.399	0.300**	0.277	0.385**	0.281**	0.290**	3.104**	3.142**	14.128	10.806**	3.716**	3.711**	7.304**	6.197**	0.821**	0.294**	11.921**	10.218**	64.734	69.138**	0.067**	0.023**	22,387.076**	20,630.707**
	(0.348 - 0.355)	(0.309 - 0.321)	(-52,742.865 - 52,743.663)	(0.295 - 0.305)	(-113,945.247 - 113,945.801)	(0.380 - 0.391)	(0.278 - 0.285)	(0.284 - 0.295)	(3.088 - 3.120)	(3.116 - 3.168)	(-2832762.431 - 2832790.688)	(10.614 - 10.998)	(3.688 - 3.744)	(3.669 - 3.753)	(7.169 - 7.440)	(6.019 - 6.375)	(0.796 - 0.846)	(0.270 - 0.317)	(11.772 - 12.071)	(10.020 - 10.416)	(. - .)	(68.860 - 69.416)	(0.065 - 0.069)	(0.021 - 0.025)	(22,228.999 - 22,545.154)	(20,402.974 - 20,858.440)
Observations	1,042,410	493,977	747,147	344,301	747,147	344,301	1,042,410	493,977	1,042,410	493,977	747,147	344,301	1,042,410	493,977	1,042,410	493,977	1,042,410	493,977	1,042,410	493,977	747,147	344,301	1,042,410	493,977	747,147	344,301
R-squared	0.145	0.143	0.163	0.169	0.275	0.179	0.138	0.135	0.120	0.138	0.143	0.139	0.071	0.079	0.100	0.091	0.100	0.049	0.115	0.103	0.174	0.143	0.120	0.069	0.120	0.109

Notes: Models also include quarter and hospital fixed effects.

© 2019 Meyers DJ et al. *JAMA Network Open.*
